# On the Need for Theoretically Guided Approaches to Possible Bilingual Advantages: An Evaluation of the Potential Loci in the Language and Executive Control Systems

**DOI:** 10.1162/nol_a_00041

**Published:** 2021-11-11

**Authors:** Esti Blanco-Elorrieta, Alfonso Caramazza

**Affiliations:** Department of Psychology, Harvard University, Cambridge, MA, USA; Center for Mind/Brain Sciences (CIMeC), University of Trento, Trento, Italy

**Keywords:** bilingual advantage, cognitive control, transfer mechanism

## Abstract

Whether a cognitive advantage exists for bilingual individuals has been the source of heated debate in the last decade. While empirical evidence putatively in favor of or against this alleged advantage has been frequently discussed, the potential sources of enhanced cognitive control in bilinguals have only been broadly declared, with no mechanistic elaboration of where, why, and how this purported link between bilingualism and enhanced language control develops, and how this enhancement transfers to, and subsequently improves, general executive function. Here, we evaluate different potential sources for a bilingual advantage and develop the assumptions one would have to make about the language processing system to be consistent with each of these notions. Subsequently, we delineate the limitations in the generalizations from language to overall executive function, and characterize where these advantages could be identified if there were to be any. Ultimately, we conclude that in order to make significant progress in this area, it is necessary to look for advantages in theoretically motivated areas, and that in the absence of clear theories as to the source, transfer, and target processes that could lead to potential advantages, an inconsistent body of results will follow, making the whole pursuit of a bilingual advantage moot.

## INTRODUCTION

During the last 15 years, the largest debate in the cognitive neuroscience of bilingualism has been whether bilingual individuals develop control mechanisms that are enhanced as compared to monolingual individuals (i.e., the bilingual advantage hypothesis). A rich body of literature has consistently reported that bilingualism enhances certain aspects of the executive function (Bialystok, 2007, 2009, 2017; Bonfieni et al., 2020; Costa et al., 2009; Zhou & Krott, 2018), such as conflict monitoring (e.g., Hofweber et al., 2016) and inhibition of irrelevant information (e.g., Costa et al., 2008; Soveri et al., 2011). However, an equally compelling body of literature has failed to find such advantages in executive control (Paap & Greenberg, 2013; Paap, Johnson, & Sawi, 2014; Paap et al., 2015, 2016; Paap, Sawi, et al., 2014; von Bastian et al., 2016; Woumans & Duyck, 2015), leading an opposing group of scientists to claim that there is no coherent evidence for a bilingual cognitive advantage (Duñabeitia et al., 2014; Duñabeitia & Carreiras, 2015; Paap & Greenberg, 2013; for an in depth review of evidence on both sides see van der Noort et al., 2019).

While the entirety of the empirical evidence and whether this evidence suffices to prove or disprove the existence of such a bilingual advantage has been thoroughly discussed, the precise source and basis for the potential formation of these additional mechanisms in bilingual individuals are severely underdeveloped. Why would bilingualism lead to cognitive advantages? What is so special about managing two languages that could give an edge in general cognitive abilities to individuals who can communicate in two languages?

So far, the theory of a bilingual advantage in cognitive control has been deceptively simple (Hartsuiker, 2015). Broadly, the claim has been that because bilinguals need to constantly monitor both languages to resolve linguistic conflict during lexical selection (e.g., Colomé, 2001; Kaushanskaya & Marian, 2007; Kroll & Stewart, 1994; Thierry & Wu, 2007), their control mechanisms are trained to a larger extent than in their monolingual counterparts. Subsequently, the additional training received within the language system generalizes to domain-general cognitive control (Bialystok, 2001), leading them to show general cognitive advantages. Within this general assertion, different researchers have tried to pinpoint the source of the advantage to varying components of cognitive control. Specifically, the different flavors of this view that have been considered are as follows.

(i)  Bilinguals constantly activate both languages, which means that they need to subsequently inhibit the nontarget language to successfully produce the target language (Abutalebi & D. W. Green, 2016; D. W. Green, 1998a; D. W. Green & Abutalebi, 2013). This experience suppressing alternative elements generalizes to the effective application of inhibition in nonverbal tasks, allowing individuals to choose the correct answer amongst competing, distracting possibilities (e.g., Simon or Flanker task; Bialystok et al., 2004).
(ii)  Bilinguals constantly deal with linguistic interference. This requires them to monitor cues in their environment that signal the intended language, and they become experts at maintaining this target language information until updated cues signal a language switch. This expertise generalizes to other situations that require conflict monitoring (e.g., congruent and incongruent trials in the Simon or the Flanker tasks; Bialystok, 2006; Costa et al., 2009; Wu & Thierry, 2017).
(iii)  Bilinguals are more efficient in using monitoring mechanisms to adjust their inhibitory control to cope with interference (i.e., a combination of (i) and (ii); Morales et al., 2013; Morales et al., 2015). The dynamic combination of both of these mechanisms leads to bilinguals being subsequently better at dealing with overall interference (Bialystok et al., 2012; Costa et al., 2009).

Although slightly different in scope, all these accounts assume that language control relies on domain-general executive control (Abutalebi et al., 2012; Blanco-Elorrieta & Pylkkänen, 2016; Craik & Bialystok, 2006; Garbin et al., 2010; cf. Calabria et al., 2019) and agree that (i) there is a locus of conflict between competing linguistic elements within the language system (source domain), (ii) that dealing with this conflict involves processes that generalize via some transfer process, and (iii) that the result of this transfer is the enhancement of general cognitive control (target domain). However, these are rather broad strokes. Currently, we lack precise characterizations of the source domain, the transfer process, and the target domain. In other words, we lack a theory that predicts what circumstances particularly engage language control, an account of how skills generalize, and a theory of cognitive control that specifies what executive functions can be improved by such generalization (Hartsuiker, 2015).

In what follows, we will address this lack of theoretical specification, and we will develop, purely theoretically, the arguments and assumptions one would have to make at different levels of the language and cognitive control systems for there to even be hypothetical room for a bilingual advantage to emerge. We will refrain from discussing behavioral and neuroimaging data, both because given the number of contradictory findings, we would risk losing ourselves in experimental details and deviating attention from the goal, and because doing so is tangential to the purpose of developing the theory behind a potential bilingual advantage (for an in-depth review of this evidence, see van der Noort et al., 2019). Thus, and specifically, we will provide several accounts of what mechanisms could be the basis for increased engagement of control in bilinguals over monolinguals at the source domain, and what assumptions one would have to make about language organization and language production to support each of these proposals. Subsequently, we will unpack the issues that could then emerge at the transfer and generalization stage. In all, the aim of this article is to redirect efforts away from empirical attempts to find a bilingual advantage that are theoretically ungrounded, and to encourage the development of detailed theories that should guide future research to find the location of a bilingual advantage (should there be one).

## POSSIBLE OPERATIONAL LOCI AT THE LANGUAGE SELECTION LEVEL

### Qualitative Differences in Language Selection as Source of the Bilingual Advantage

The first possibility that we will consider is one that relates to the claims that have been previously proposed; that is, one where the relationship between bilingualism and cognitive control processes emerges from the mechanisms devised to solve across-language competition at the lexical selection level.

These accounts assume that there is an inherent difference in the way lexical selection is achieved in the bilingual case that is distinct from the way in which the same purpose is accomplished in monolingual individuals. Fundamentally, this means that the mechanism used to select equivalent elements across languages is qualitatively distinct from the way comparable elements are picked from within a single language. According to models that posit different language architectures for bilingual and monolingual individuals, the key ingredient present for bilingual communication but absent in monolingual communication is categorical inhibition of a full system (i.e., the nontarget language). Succinctly, these theories propose that inhibition is applied to nontarget translation equivalents to solve competition amongst lexical alternatives and enable the selection of the target lexical element (originally the Inhibitory Control Model, D. W. Green, 1998a, 1998b; subsequently developed in Abutalebi & D. Green, 2007; Abutalebi & D. W. Green, 2016; D. W. Green & Abutalebi, 2013).

There are a number of unresolved questions with this proposal, however. First, if one argues inhibition to be the most efficient mechanism to deal with, and choose from, competing representations, presumably this should be the default mechanism for choosing amongst competing lexical representations regardless of the number of languages a person knows. In other words, this should also be the default mechanism used by every individual to choose between equivalent options, whether these come from within a language (e.g., to choose amongst synonyms, different registers [Declerck et al., 2020], dialects [Kirk et al., 2018; Vorwerg et al., 2019], etc.), or across languages (e.g., translation equivalents). Making this mechanism general though, could nullify it as a viable option for the source of the bilingual advantage.

There are three ways around this issue to keep inhibition as the source of the advantage. One is to address these concerns by developing a principled argument as to why only bilinguals use this mechanism despite its supremacy as a lexical selection mechanism, and a proposal regarding the point in learning a second language where bilinguals acquire this additional device. Such a theory has not been developed, and we are currently unable to provide a solid argumentation to advocate for this possibility. Thus, we will leave this possibility aside until such a detailed proposition is successfully developed.

The second possibility to keep inhibition as the source of the advantage and avoid answering the previous questions, is to assume that inhibition is in fact the tool used by both monolinguals and bilinguals to deal with conflicting representations, but that there is more competition from across language elements as compared to within language elements, which leads to a sharper tuning of this mechanism in bilinguals. This workaround although compelling at face value, comes with its own set of issues. If inhibition is applied to the whole nontarget language as inhibition-based models of lexical selection suggest (Abutalebi & D. W. Green, 2016; D. W. Green, 1998a; D. W. Green & Abutalebi, 2013), once inhibition is applied at the highest level of selection, then competition would no longer emerge throughout the rest of the linguistic system during production, as everything that could potentially cause competition would have been suppressed to begin with. Namely, once inhibition to the nontarget language has been applied, competition in the production system is reduced to within language competitors, equalizing demands on monolingual and bilingual lexical selection. Hence, to make this possibility work, one would have to adopt the following postulates: (i) the language system operates on the basis of inhibition, (ii) executive control is intrinsically and definitionally a process of suppression, and (iii) the first step of suppression is so strong that it is enough to cause an advantage in and of itself, despite it facilitating language selection throughout the rest of the language production system. However, there is currently no evidence suggesting that the first step of inhibition could be that overarching, and the general consensus in the literature is that executive control involves much more than just inhibition; hence it does not really follow that from a single step of inhibition a general advantage in cognitive control would emerge.

The third possibility to maintain inhibition at the center of the advantage, and avoid answering the first set of questions and the adoption of these ungrounded tenets, is to assume that inhibition is in fact the tool used both by monolinguals and bilinguals during lexical selection, that there is more competition from across than within language elements, but that the inhibition to solve this competition is applied at the individual word level as opposed to at the whole language level (as proposed in the distinction between local vs. global inhibition; e.g., Branzi et al., 2016; Guo et al., 2011). This would mean that it is not the case that once inhibition is applied at the whole language level, competition is eliminated throughout the language system, but rather, that inhibition needs to be applied and reapplied at every point of the discourse, making it a recurrent process that would improve inhibitory skills in bilingual individuals. However, while the application of inhibition at each element independently can be useful to explain laboratory tasks where participants are required to randomly switch languages in interleaved trials, the interconnectedness of speech and planning of linguistic structures above the single-word unit during natural communication makes it challenging to extend this proposal to such contexts. Presumably, in the process of natural utterance planning, an individual would either inhibit every element in the nontarget language, or would inhibit none and allow for the free selection of elements of either language to enable rampant code-switching, but it is unclear what circumstances could dominate the communicative context to force speakers to inhibit each word in turn and apply and reapply inhibition at each step of the discourse. Thus, although this possibility could perhaps work within the context of experimental tasks, it does not seem to stand the scrutiny of generalization to natural communication. Since the advantage in bilingual individuals needs to necessarily emerge from a mechanism applied during natural language use, we are also unable to place this mechanism as the source of a real bilingual advantage.

To recapitulate, we have evaluated four different possibilities that place inhibition as the distinctive feature of bilingual language selection and central locus of the advantage of bilingual individuals. Although all appealing at a surface level, once exposed to deeper examination, qualitative differences between monolingual and bilingual language selection do not seem to satisfy the premises required for them to lead to an enhancement of cognitive mechanisms in bilinguals. Consequently, we will now proceed to evaluate alternatives that are not based on categorical differences between monolingual and bilingual language organization but rather on quantitative differences between the two.

### Quantitative Differences in Language Organization

If one assumes that both monolingual and bilingual language systems work on the same principles (e.g., Blanco-Elorrieta & Caramazza, 2021), in order for the bilingual advantage to emerge within the language system, two premises ought to be true. First, there needs to be a lexical selection process that is shared across monolingual and bilingual individuals that requires the involvement of executive control. Second, this process needs to be more often or more strongly recruited in bilinguals than monolinguals such that this increased use ultimately sharpens these control abilities in bilinguals. Although currently there is no evidence to suggest that in fact executive control needs to mediate lexical selection, and recent models suggest that levels of activation could suffice to successfully pick target elements (e.g., Blanco-Elorrieta & Caramazza, 2021), we will develop the scenarios under which those assumptions could theoretically lead to bilingual advantages.

One possibility to satisfy both of these premises is to adopt Roelofs’ notion of competition (Roelofs, 1992), which asserts that a lexical choice becomes harder the more similar two competing alternatives are, and assume that executive control is needed to untie this similarity and make a fine-grained choice amongst remarkably similar options. In the case of monolingual individuals, this competition is at its highest for synonyms or near-synonyms (Lachman, 1973; Levelt et al., 1991; Peterson & Savoy, 1998; Vitkovitch & Tyrrell, 1995). Bilingual individuals additionally face these hard choices when presented with cognates and translation equivalents (Hartsuiker & Notebaert, 2010; Ivanova & Costa, 2008; Marian & Spivey, 2003; Sarkis & Montag, 2021), particularly given that both languages are constantly coactivated (e.g., Blumenfeld & Marian, 2013; Gullifer et al., 2013; Martin et al., 2009), which exponentially multiplies their need to rely on this type of executive function if lexical selection would in fact be mediated by executive control.

Additionally, if one assumes a model of lexical selection where there is independent, cascading activation (e.g., Caramazza, 1997), this increase in harder choices for bilinguals would not be reduced to the lexical level, but rather it would ripple through and reproduce in the rest of the linguistic levels. Thus, while monolinguals are limited in terms of the opportunities for having competing phonemes, lexical items, and syntactic formulations to certain synonyms, register or dialectal variations, bilinguals would be faced with quasi-equivalent elements at almost every point in the discourse across all of these levels.

Consequently, this proposal could satisfy both theoretical requirements in that it acknowledges that both monolingual and bilingual speakers use the same mechanisms for the same kinds of qualitative choices, but recognizes that the sheer number of these types of choices a bilingual has to face are exponentially higher, and this larger number of choices requiring control that bilinguals have to make constitutes the locus of increased control during bilingual language selection. The prediction that would naturally follow is that bilinguals would in principle be more efficient at lexical selection, both in their first and their second language. However, it is possible that this improved retrieval would be behaviorally undetectable, because bilingual individuals would continue to suffer from the increased competition from the second language that leads to sharper tuning of executive control to begin with. Importantly, this proposal hinges upon empirically showing that (i) the more similar two elements are, the harder selection becomes (Roelofs, 1992), and (ii) the selection process between these highly-similar alternatives requires the mediation of executive control. Although these two tenets are so far empirically unproven, they could at least theoretically work.

However, even if one were to believe in the correctness of those two assumptions, there is a premise that is still missing from this proposal, and that is that there ought to be some external constraint determining which is the correct and which is the incorrect option amongst the competitors for one to argue that some control is required to mediate these choices. This requirement follows because if either one of the quasi-equal candidates were an equally valid choice for production, elements could be stochastically chosen without any need for mechanisms making fine-grained selections.

One possibility to straightforwardly implement this external constraint is through the linguistic demands posited by the interlocutor’s linguistic profile (i.e., the need to choose the option that the interlocutor will understand). In what follows, we will evaluate the three conversational scenarios that have been previously delineated for bilinguals—dense code-switching contexts, dual-language contexts, and single-language contexts (D. W. Green & Abutalebi, 2013)—and assess whether the requirement for executive function to mediate selection would uphold in each of them.

(a)  In dense code-switching contexts, both languages can be used with all interlocutors, as everyone shares the linguistic profile of the speaker. This allows bilinguals to select the production language freely, without needing to constrain it based on audience considerations. Consequently, there would be no need to make targeted choices that executive control needs to mediate. In other words, even if there truly were a bilingual advantage, and the source of this advantage would in fact lie in the quantity of executive-control mediated choices bilinguals need to make, bilinguals who are most frequently immersed in this type of context would not develop such advantages because the freedom to choose any available element would mean that they do not often need to make these fine-grained choices.
(b)  In dual-language contexts both languages are also used, but each of them is used with different individuals (i.e., each interlocutor or group of interlocutors exclusively understands one of the possibilities available to the speaker). Given the assumption that executive control mediates the selection amongst highly similar choices, it would follow that such control would be required in these scenarios to ensure that at each point in the discourse the element that the interlocutor will understand is selected from the pool of competing candidates. Thus, this context could fulfill all the premises for it to lead to the development of a bilingual advantage provided that the assumption that executive control mediates this kind of selection holds true.
(c)  Last, in single-language contexts, all interlocutors understand only one of the languages available to the speaker. Hence, the premise is met that an external factor will guide possible choices in this context. However, and critically, the assumption is that executive control will mediate candidate selection to untie and choose between equally available, highly similar options. Given sufficient background context or experience with a given individual, the activation levels for the language that is not shared with the listener will be much lower than those for the shared language. Consequently, the two choices will not be similarly available for selection, which means that there would no longer be a need for executive control to mediate those choices. An exception in this context could be the case of highly unbalanced bilinguals required to produce their second language. Since native language elements will have higher activation levels by default, increased activation of their second language when attempting to produce said language could balance activation levels, leading to equally available candidates and the subsequent need for mediation of executive function. In all, even though theoretically all the requirements to develop a bilingual advantage could be met in the single-language context, it would seem that in practice only very specific combinations of language proficiencies and context would actually satisfy such premises in this context.

Importantly, bilingualism being a dynamic life experience, bilingual individuals do not categorically belong to one of these three contexts but rather they will find themselves more or less often in each of these communicative scenarios depending on a variety of social backgrounds and experiences. Here we argue, in line with previous proposals (Blanco-Elorrieta & Pylkkänen, 2018; Kaan et al., 2020), that this experience will modulate the extent to which they may be more or less likely to develop the purported advantages in the source domain, if both required premises—(i) that a lexical choice becomes harder the more similar two competing alternatives are, and (ii) that executive control is needed to untie this similarity and make a fine-grained choice amongst highly similar options—hold.

## THE ISSUE OF TRANSFER AND GENERALIZATION

The previous section unpacked the theoretical possibilities for the source of a potential bilingual advantage within the language system. However, even if the two assumptions required for our best proposal would empirically hold, that would still only cover the grounds for where additional training in bilinguals could emerge. To make informed predictions about whether this would translate into a bilingual advantage in general cognitive control, we still need a characterization of the relationship between executive control as applied in language production and general-purpose cognitive control, and as well an understanding of cognitive control generally, which in and of itself has been elusive to characterize. As Ridderinkhof et al. (2011) point out, “perhaps due to its descriptive rather than mechanistic conceptualization, cognitive control has long remained an intractable concept” (p. 174). That is to say—we do not exactly know how to measure cognitive control and what even cognitive control is, beyond an enumeration of functions that may fall under its umbrella (e.g., inhibition, switching, and monitoring).

In the absence of evidence that illuminates the true nature of cognitive control, there are two main possibilities to consider. On the one hand, it could be that there is a unique central engine of executive control, which is blind to the specific task that it is applied to. Thus, this mechanism would be engaged in every task that requires executive control, regardless of the specifics of the task. Applied to the context of language, this proposal would align with accounts where language control relies on domain-general cognitive control (Abutalebi et al., 2012; Blanco-Elorrieta & Pylkkänen, 2016; Craik & Bialystok, 2006; Declerck et al., 2021; Garbin et al., 2010; cf. Calabria et al., 2019). Adopting this notion of a unique executive control mechanism would mean that improvement in one task would automatically result in enhancements in every other task that also requires cognitive control, since both tasks would rely on the same unique executive function machinery. Adopting such a vision of executive function would predict that ultimately, if both language use and violin playing required the engagement of this central cognitive control, training executive function during language production (e.g., by being bilingual) would automatically imply improvement in violin playing.

Alternatively, it could be that there is a central instance of executive control that is then exercised specifically within the context of particular applications (i.e., both language use and violin playing require executive function, but critically they constitute fundamentally distinct applications of it). This proposal aligns instead with accounts that suggest that there is such a thing as language-specific control (e.g., Abutalebi et al., 2008; Calabria et al., 2012). If this were the case, then one would have to determine the extent to which training in one specific application of cognitive function transfers and generalizes to the main executive function engine, such that it could subsequently improve the rest of the specialized applications of such control. In consequence, under this account, there would need to be a detailed account of how transfer works, and how exactly language practice would lead to improvements in the rest of the tasks that require cognitive control.

Importantly neither the transfer nor the generalization issue are restricted to the bilingual advantage, but rather they are a recurrent topic of discussion in research that tries to unpack cognitive benefits derived from a range of other tasks. In different historical waves, there have been attempts to establish direct causal benefits to one cognitive domain from practice in another task, including, for example, whether music practice improves math skills or whether reading ability improves memory. In recent years, for instance, there has been an explosion of research on the potential benefits of video-game playing, with empirical results showing that video-game players outperform non–video-game players on tests of attention control, working memory, and executive control (Blacker & Curby, 2013; Cain et al., 2012; Castel et al., 2005; Chisholm & Kingstone, 2012; Colzato et al., 2013; C. S. Green & Bavelier, 2003, 2006, 2007; McDermott et al., 2014), only to ultimately conclude under more thorough examination that in most cases, training only improves the specific skill one is training (e.g., Ericsson & Charness, 1994; Gobet et al., 2001; Gobet, 2015; Hambrick et al., 2017; Powers et al., 2013; Redick et al., 2017; Sala et al., 2018; Sala & Gobet, 2019; Schellenberg, 2020; Simons et al., 2016; Unsworth et al., 2015).

The consequences of this finding in other cognitive domains are nontrivial. First, it would suggest that in fact there is no single, unified executive function that is equally applied to every task that requires cognitive control, and consequently one would have to explore possible routes for transfer and generalization. Second, if across other domains of cognition, the conclusion is that there is no real generalization of skills, one would either have to argue an extremely convincing case for why language would be different than the rest of the cognitive skills, or the search for advantages in cognitive control would have to be limited to the exact skill that the theory of language one ascribes to posits as the root of the advantage at the source domain. It follows then that looking for a bilingual advantage necessitates a clearly articulated theory of language selection and a characterization of the specific loci for where the advantage could lie. In this paper, we conducted that exercise by carefully considering the prevalent models of lexical selection, and we conclude that the only possible source for an advantage at the language level that is theoretically possible is one where the lexical choices become harder the more similar two competing alternatives are (Roelofs, 1992), and where executive control is needed to untie this similarity and make a fine-grained choice amongst highly similar options. In the absence of empirical evidence supporting these two tenets though, one resorts back to models where the highest level of activation is sufficient for lexical selection in monolingual and bilingual individuals (e.g., Blanco-Elorrieta & Caramazza, 2021), which would lead to no theoretical grounds for an advantage to emerge at the source domain.

## CONCLUSION

The study of a potential bilingual advantage in cognitive function has dominated the field of bilingual research in the last decades, yet the empirical basis for this hypothesis remains shaky (Hartsuiker, 2015; Paap et al., 2016). These inconsistencies may emerge to a large extent because this endeavor has involved attempts to find advantages in every subcomponent of executive control without a particular theory of where these potential advantages would emerge from or how and why they would have transferred to general cognitive control.

Here, we laid out different possibilities for the potential loci of the bilingual advantage at the source domain and evaluated what assumptions one would have to make about the lexical system of bilingual individuals for each of them to hold. After careful consideration, we are left with only one possibility that could theoretically work, even though it still requires assumptions that are not yet empirically proven, and would thus require experimental confirmation. Further, we find that even if these were to hold, the convergent empirical evidence from other fields suggests that the generalization could only really apply to the exact same skill in another domain. In all, we advocate that in order for progress to be made in this field, we need theoretically guided approaches that rely on detailed accounts of language selection and transfer to cognitive control. Even though the empirical evidence so far may more convincingly support postulates that there is in fact no cognitive advantage for bilinguals (Duñabeitia et al., 2014; Duñabeitia & Carreiras, 2015; Paap & Greenberg, 2013; Paap, Johnson, & Sawi, 2014, 2015, 2016; Paap, Sawi, et al., 2014; von Bastian et al., 2016; Woumans & Duyck, 2015), the evidence showing bilingual advantages in some contexts and tasks (Bialystok, 2007, 2009, 2017; Bonfieni et al., 2020; Costa et al., 2009; Zhou & Krott, 2018) merits consideration of the fundamental claim. However, we may need to take a step back and develop and reassess the theory from which these advantages could stem in order to be able to develop the targeted approaches that may enable us to find them.

## FUNDING INFORMATION

Alfonso Caramazza, Harvard University, Award ID: Provostial fund.

## AUTHOR CONTRIBUTIONS

**Esti Blanco-Elorrieta**: Conceptualization: Lead; Writing – original draft: Lead; Writing – review & editing: Equal. **Alfonso Caramazza**: Conceptualization: Equal; Resources: Lead; Supervision: Lead; Writing – review & editing: Equal.

## TECHNICAL TERMS

Bilingual advantage hypothesis: The idea that as a consequence of life-long bilingualism, bilingual individuals develop improved cognitive skills.
Executive control: The set of cognitive processes that are necessary for the cognitive control of behavior, such as selecting and successfully monitoring behaviors that facilitate the attainment of chosen goals.
Bilingual advantage: An advantage in various aspects of executive control including attention, inhibition of nonrelevant information, and conflict resolution, derived from the experience of being a bilingual individual and having to constantly monitor and control two languages.
Cognitive control: The ability to focus on information that is currently relevant to a particular goal, which enables the selection of a behavior that is accepted as appropriate and reject a behavior that has been deemed inappropriate.
Inhibitory control: The suppression of goal-irrelevant stimuli and behavioral responses.
Dual-language context: A conversational context in which one language is utilized with at least one interlocutor and a different language has to be utilized with at least one other interlocutor.
Dense code-switching context: A conversational context in which two languages are used, but all interlocutors in the conversation understand both languages, thus imposing no constraints on language choice.
